# Sarcopenia is associated with prognosis in patients with esophageal squamous cell cancer after radiotherapy or chemoradiotherapy

**DOI:** 10.1186/s12876-022-02296-9

**Published:** 2022-04-30

**Authors:** Junchao Qian, Youjiao Si, Ke Zhou, Yu Tian, Qisen Guo, Kaikai Zhao, Jinming Yu

**Affiliations:** 1 Department of Radiation Oncology, School of Medicine, Shandong University, Shandong Cancer Hospital and Institute, Shandong First Medical University and Shandong Academy of Medical Sciences, Jinan, 250117 Shandong People’s Republic of China; 2 Department of Radiology, Shandong Cancer Hospital and Institute, Shandong First Medical University and Shandong Academy of Medical Sciences, Jinan, 250117 Shandong People’s Republic of China; 3 Department of Radiation Oncology, Shandong Cancer Hospital and Institute, Shandong First Medical University and Shandong Academy of Medical Sciences, Jinan, 250117 Shandong People’s Republic of China; 4 Hefei Cancer Hospital, Anhui Province Key Laboratory of Medical Physics and Technology, Institute of Health and Medical Technology, Hefei Institutes of Physical Science, Chinese Academy of Sciences, Hefei, 230031 People’s Republic of China

**Keywords:** Esophageal squamous cell cancer, Sarcopenia, Body composition, Chemoradiotherapy, Prognosis

## Abstract

**Background:**

This study aimed to determine the prognostic value of the sarcopenia on the progression free survival (PFS) and overall survival (OS) of esophageal squamous cell cancer (ESCC) patients who received radiotherapy (RT) or chemoradiotherapy (CRT).

**Methods:**

Data on clinicopathological characteristics and nutritional parameters were analyzed and correlated with PFS and OS, retrospectively. Skeletal muscle, subcutaneous, visceral and total fat tissue cross-sectional areas were evaluated on CT images at the midpoint of the 3rd lumbar vertebrae. A total of 213 patients were enrolled in this study.

**Results:**

Sarcopenia was significantly associated with subcutaneous fat content. The univariate analysis demonstrated that OS was superior in patients with non-sarcopenia, non-alcohol, NRI ≥ 100, albumin ≥ 40 g/L, TATI > 83.0, SATI > 27.8, VATI > 49, non-anemia, cervical and upper-thoracic ESCC, T stage 1–2, N stage 0–1 and TNM stage I–II. In the multivariate analysis, sarcopenia, albumin, N stage and TNM stage were identified as independent prognostic factors of survival. This study demonstrated that sarcopenia was related to worse PFS and OS in patients with ESCC who received RT or CRT.

**Conclusions:**

Sarcopenia is considered to be a useful predictor in patients with ESCC who received RT or CRT. This study also provided a conceptual basis for further prospective research on the application of the sarcopenia for patients receiving RT or CRT for intermediate- and advanced-stage ESCC.

## Introduction

The esophageal cancer is one of the leading causes of mortality among the digestive tract cancers [1]. The main histology type is Esophageal Squamous Cell Carcinoma (ESCC), which accounts for 90% of esophageal cancers worldwide [2]. Since most patients are diagnosed in advanced stages and unable to undergo surgery, the reference treatment for patients with ESCC is chemoradiotherapy (CRT) [2, 3]. However, the prognosis remains disappointing with 5 years of survival rates following CRT or CT of around 30% [4].

Most of patients with advanced ESCC are affected by cancer-related cachexia, which results in wasting of skeletal muscle mass with or without loss of fat mass [5]. Meanwhile, CRT treatment-related dysphagia, nausea or vomiting also induce the deterioration of cachexia, leading to a change in body composition and malnutrition [6, 7]. Moreover, nutritional support was reported to improve weight gain, overall survival rate and quality of life in patients [1, 8, 9]. Hence, identifying convenient and effective nutritional prognostic factors before treatment is of great importance.

Sarcopenia reduces physical activity, which leads to decreased energy and poor prognosis. To this end, the important role of sarcopenia has reached consistent recognition and the Asian Working Group for Sarcopenia (AWGS) 2019 consensus was developed [10]. Large amounts of evidence indicate that sarcopenia independently correlate with poorer OS in malignant tumors, including head and neck cancer [11], lung cancer [12, 13], colorectal cancer [14, 15], gastric cancer [16], and renal cell cancer [17]. Sarcopenia is associated with mortality, and lower OS in patients with ESCC. Esophagus is an important digestive organ, the occurrence of esophageal tumor seriously affects the nutritional status of patients, and then reduces the prognosis of patients. Some studies also suggested thatsarcopenia is associated with impaired OS after surgery for oesophageal cancer [1, 18], however, the prognostic value of sarcopenia for patients with ESCC who received RT or CRT has not been demonstrated yet.

In this study, we investigate sarcopenia before treatment and its association with nutritional status, and survival of patients, and thus evaluated the prognostic value of sarcopenia for survival through univariate and multivariate analyses in ESCC patients who received RT or CRT.

## Materials and methods

### Patients

**Fig. 1:**
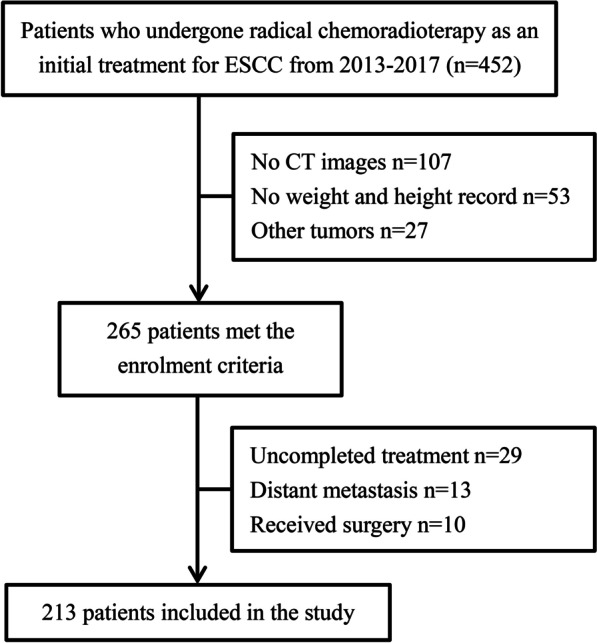
Consort diagram. *N* number of patients

The records of all 213 ESCC patients were confirmed by pathology and collected from Shandong Cancer Hospital between June 2013 and December 2017 in accordance with the STROBE statement. Patients were included in this study if they had RT or CRT. The exclusion criteria were as follows: (1) history of other malignancies, (2) incomplete medical records (such as no electronic CT image data, height or weight information etc.), (3) distant metastasis. A consort flow diagram of patient selection is shown in Fig. 1. The 7th edition of the American Joint Committee on Cancer TNM staging system was used to determine the clinical or pathological stage. This retrospective study was approved by the Ehics Committee of Shandong Cancer Hospital and Institute. Formal written informed consent was waived by the Ethics Committee of Shandong Cancer Hospital and Institute owing to its retrospective study design, and all data were kept confidential.

### Treatment

Patients received the same radiotherapy with photons (6 MV) to a total dose of 50–70 Gy (median 60 Gy) with 1.8–2.0 Gy/day, 5 days per week. 63 patients received RT, 112 patients received concurrent CRT and 38 patients received sequential CRT. 150 patients receiving chemotherapy treated with platinum-based chemotherapy (median of 3 cycles, range 1–6 cycles).

### Image analysis of subcutaneous and visceral fat mass and skeletal muscles

**Fig. 2:**
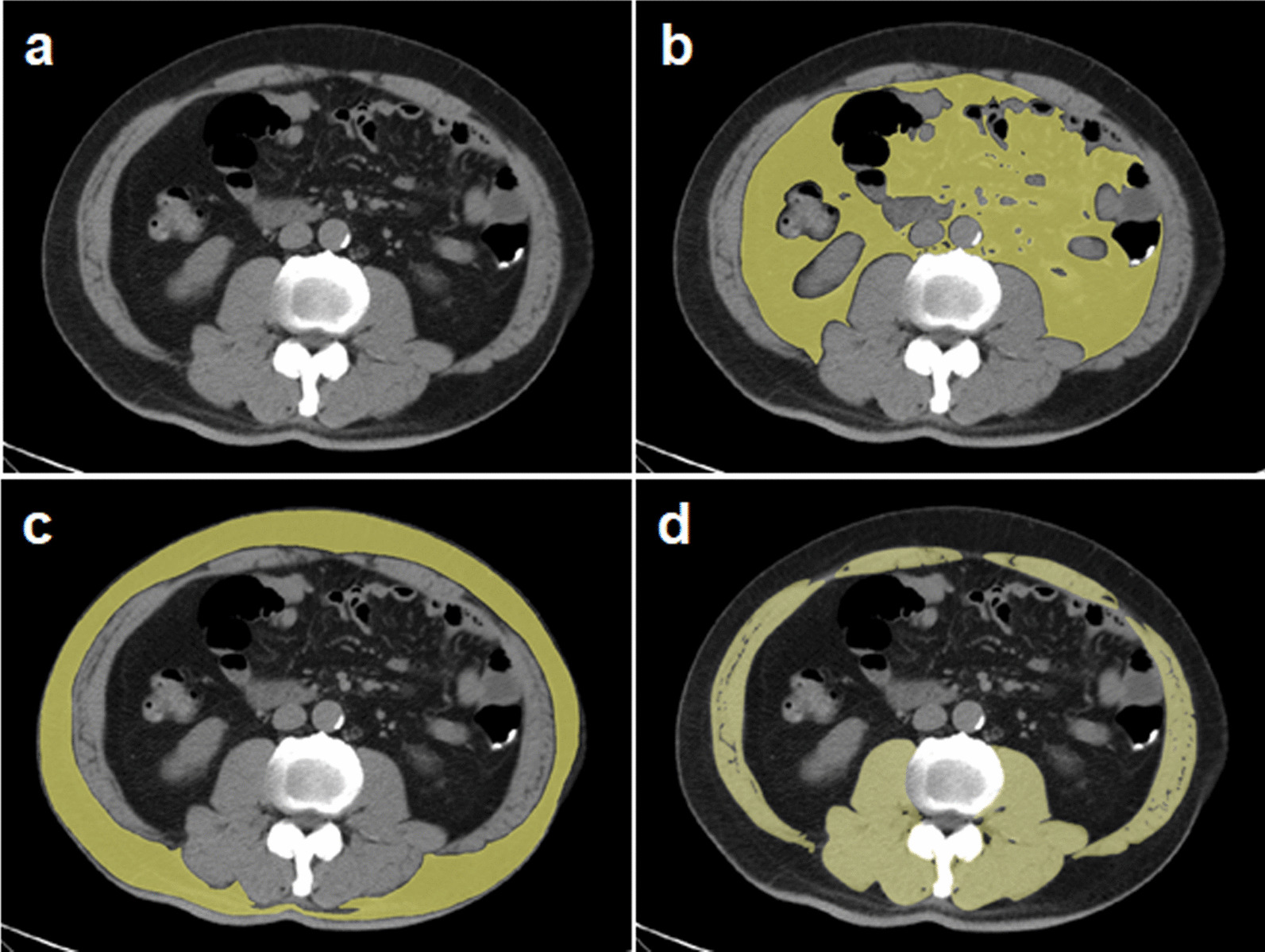
Body composition measurements using computed tomography at the level of third lumbar vertebrae. (**A**) Original computed tomography images for measurements. (**B**) Delineation of visceral fat area with a threshold of − 150 to − 50 HU. (**C**) Delineation of subcutaneous fat area with a threshold of − 190 to − 30 HU. (**D**) Delineation of total skeletal muscle area with a threshold of − 29 to + 150 HU

In all patients, computer tomography scans were acquired less than 2 weeks prior to RT or CRT. Skeletal muscle, subcutaneous fat, visceral fat and total fat area were measured at the midpoint of the third lumbar vertebrae (L3) as described previously (Fig. 2A–D) [7, 19, 20]. L3 was chosen because tissue in this region has been found to be most correlated with whole body composition [7, 21]. In briefly, images from computed tomography (Somatom AR.C; Siemens, Erlangen, Germany) scans on participants in the supine position were used for analysis. Visceral and subcutaneous fat area were calculated using a software OsiriX (Pixmeo, Geneva, Switzerland) [1, 21] and with a threshold within − 190 to − 30, − 150 to − 50 Hounsfield units (HUs), respectively. The Hounsfield unit thresholds of − 29 to 150 were then set for skeletal muscles including the rectus abdominis, transverse abdominal, psoas, paraspinal, internal and external oblique muscles. Regions of the subcutaneous, visceral fat and skeletal muscles area was defined by tracing its contour on each scan, and manual corrections were performed when necessary [1, 21]. Sarcopenia has been defined as a skeletal muscle index (SMI) of ≤ 41 cm^2^/m^2^ for women and ≤ 53 cm^2^/m^2^ in case of a body mass index (BMI) of ≥ 25 kg/m^2^ or ≤ 43 cm^2^/m^2^ in case of a BMI of < 25 kg/m^2^ for men [7].

### Clinical definitions

We calculated BMI (kg/m^2^) values by based on a direct measurement of weight (kg)/height^2^ (m^2^) at diagnosis, we divided patients into obesity (BMI ≥ 24) and no obesity groups (BMI < 24) according to previous study [22]. Anemia was defined as serum hemoglobin (Hb) levels ≤ 12 g/dL in women and ≤ 13 g/dL in men according to Chinese criterion [23]. Serum albumin was divided into normal and low albumin groups according to the normal value defined by laboratory in our hospital. Patients in this cohort were divided into two groups based on median tumor length (≤ 4 cm and > 4 cm). The age was categorized according to previous study [24]. Nutritional Risk Index (NRI) was then calculated using the following formula: NRI = (1.519 × albumin g/dL) + 41.7 × (present body weight/ideal body weight) [25]. The ideal body weight was calculated using the Lorentz formula [26]. The nutritional status of each patient was stratified according to the calculated NRI: NRI score ≥ 100: no risk; < 100: have risk [8].

### Follow-up

OS was computed from the day of the pathological diagnosis of ESCC until death or the last follow-up. The second endpoint was PFS, defined as the time between day 1 and the first event of local failure, metastatic recurrence, progression, or death. Patients lost to follow-up were “censored” at the date of the last clinic visit.

Patients were followed up in the hospital once every 3 months for the first 2 years, once every 6 months for the third year, and once yearly from the fourth year. During the re-examination, the patient received blood routine examination, liver and kidney function, tumor markers and other laboratory tests, as well as chest and abdomen computed tomography examination. Bone emission computed tomography was performed every 6 months.

### Statistical analysis

SPSS 22.0 (IBM Corporation, Armonk, NY, USA) was used for statistical analysis. A χ^2^ test or Fisher’s exact test was performed for comparing patient baseline characteristics. PFS and OS were calculated using Kaplan–Meier analysis method and log-rank tests. The Cox regression model was used for univariate and multivariate analysis. After the univariate analysis, those variables with *p* < 0.2 were taken part in the multivariable analysis and Cox stepwise forward regression analysis and Wald test were conducted. *p* < 0.05 was considered statistically significant.

## Results

### Patient characteristics

**Fig. 3:**
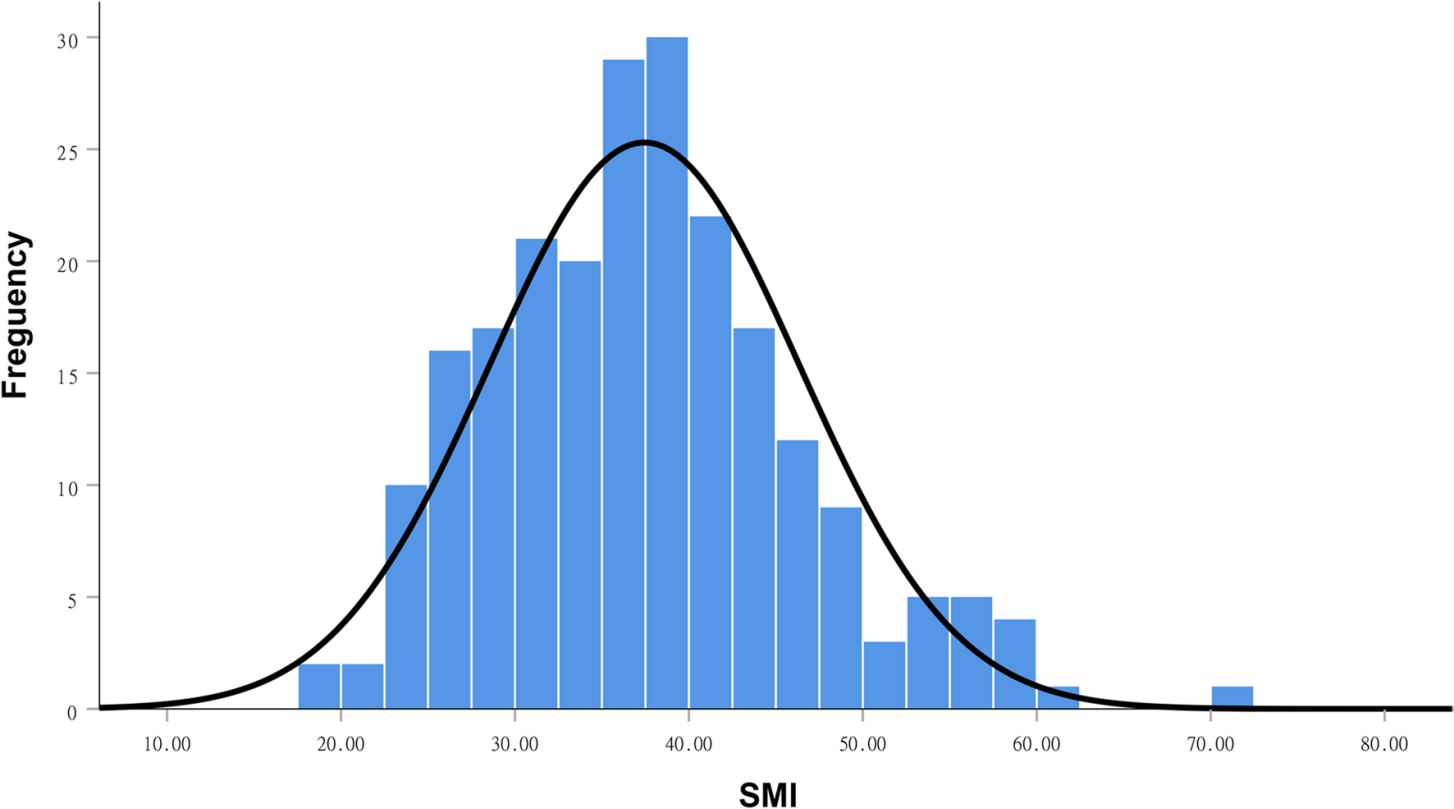
The SMI exhibited a normal distribution

A total number of 213 patients with ESCC undergoing CRT or CT were assessed. Baseline characteristics of all included patients are shown in Table 1. The study group consisted of 160 men (75.1%) and 53 women (24.9%), from 44 to 85 years (median 67). 114 patients (53.5%) had a history of smoking. 100 patients (46.9%) in our cohort had a history of alcohol use. The most common location of primary tumors was the middle second of the thoracic esophagus (40.4%). The length of tumor lesions ranged from 1.0 to 10.5 cm (median 4.5). The median BMI was 22.5 kg/m^2^ (range 14.5–31.2 kg/m^2^). The SMI exhibited a normal distribution (Fig. 3), and the mean SMI was 37.3 ± 8.7. The number of patients in the sarcopenia group and non-sarcopenia group was 170 (79.8%) and 43 (20.2%), respectively.

**Table 1:** Baseline characteristics

Characteristic	Non-sarcopenia	Sarcopenia	*p* value
	n = 43, 20.2%	n = 170, 79.8%	
Age, years			0.163
> 65	21	103	
≥ 65	22	67	
Gender			0.064
Male	37	123	
Female	6	47	
Smoking			0.307
Yes	26	88	
No	17	82	
Alcohol			0.781
Yes	21	79	
No	22	91	
BMI, kg/m^2^			0.145
≥ 24	19	55	
< 24	24	115	
NRI			0.234
≥ 100		38	137
< 100		5	33
Anemia			0.325
Yes	12	61	
No	31	109	
Albumin			0.306
< 40	7	40	
≥ 40	36	130	
SATI (cm^2^/m^2^)			**0.009**
≤ 27.8	14	93	
> 27.8	29	77	
VATI (cm^2^/m^2^)			0.633
≤ 49.0	20	86	
> 49.0	23	84	
TATI (cm^2^/m^2^)			0.133
≤ 83.0	17	89	
> 83.0	26	81	
Location			0.063
Neck/upper	25	72	
Middle/lower	18	98	
Length (cm)			0.116
≤ 4	17	90	
> 4	26	80	
Treatment strategies			0.678
RT	8	55	
CRT	36	114	
TNM stage			0.871
I–II	16	61	
III–IV	27	109	
T stage			0.961
T1–2	9	35	
T3–4	34	135	
N stage			0.683
N0–1	14	61	
N2–3	29	109	

*P* value highlighted in bold indicated *p* < 0.05

### Sarcopenia and prognosis of ESCC

**Fig. 4:**
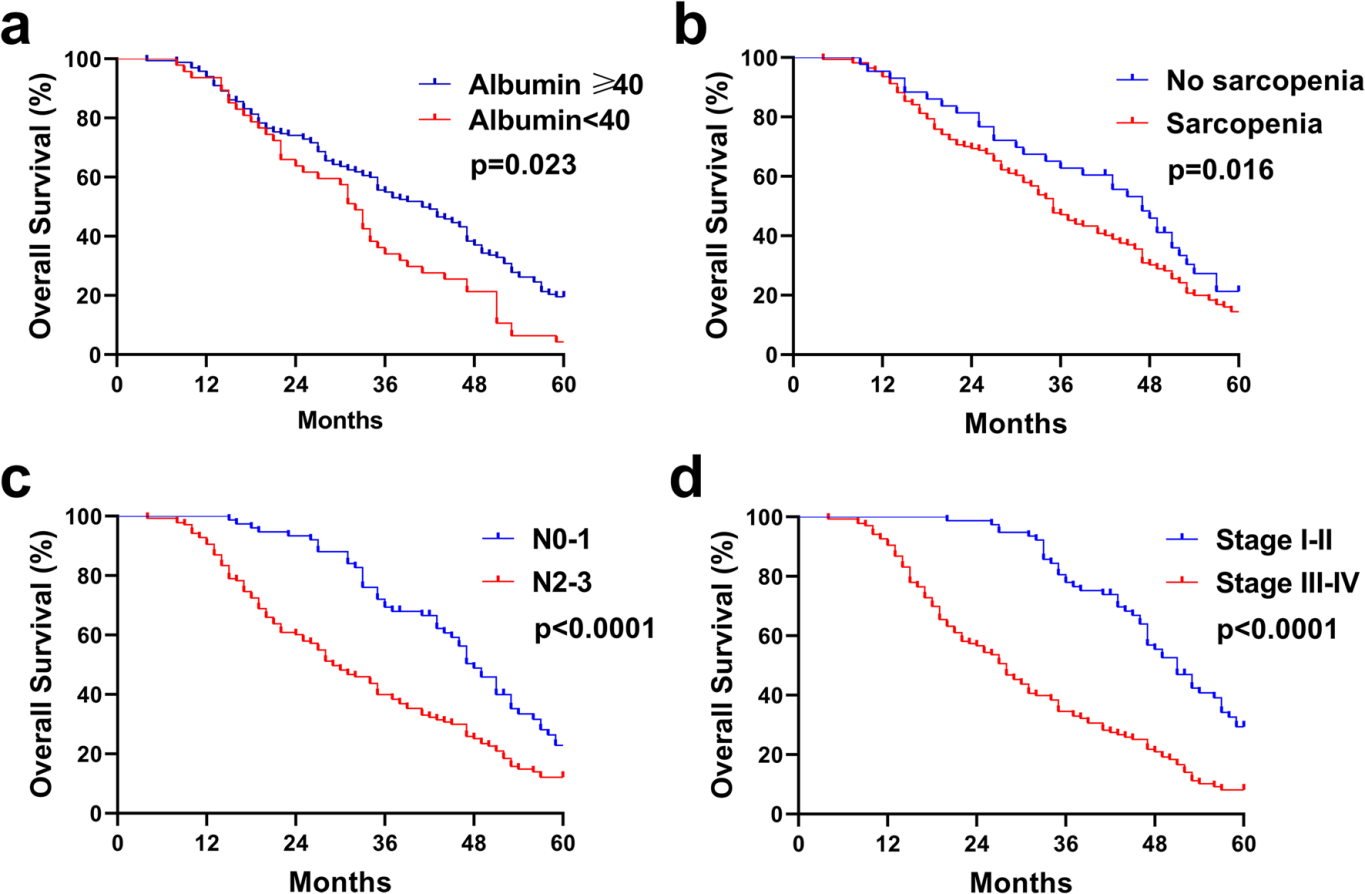
Kaplan–Meier survival curves. (**A**) OS of patients with albumin ≥ 40 or < 40. (**B**) OS of patients according to sarcopenia. (**C**) OS of patients according to N stage. (**D**) OS of patients according to TNM stage

**Fig. 5:**
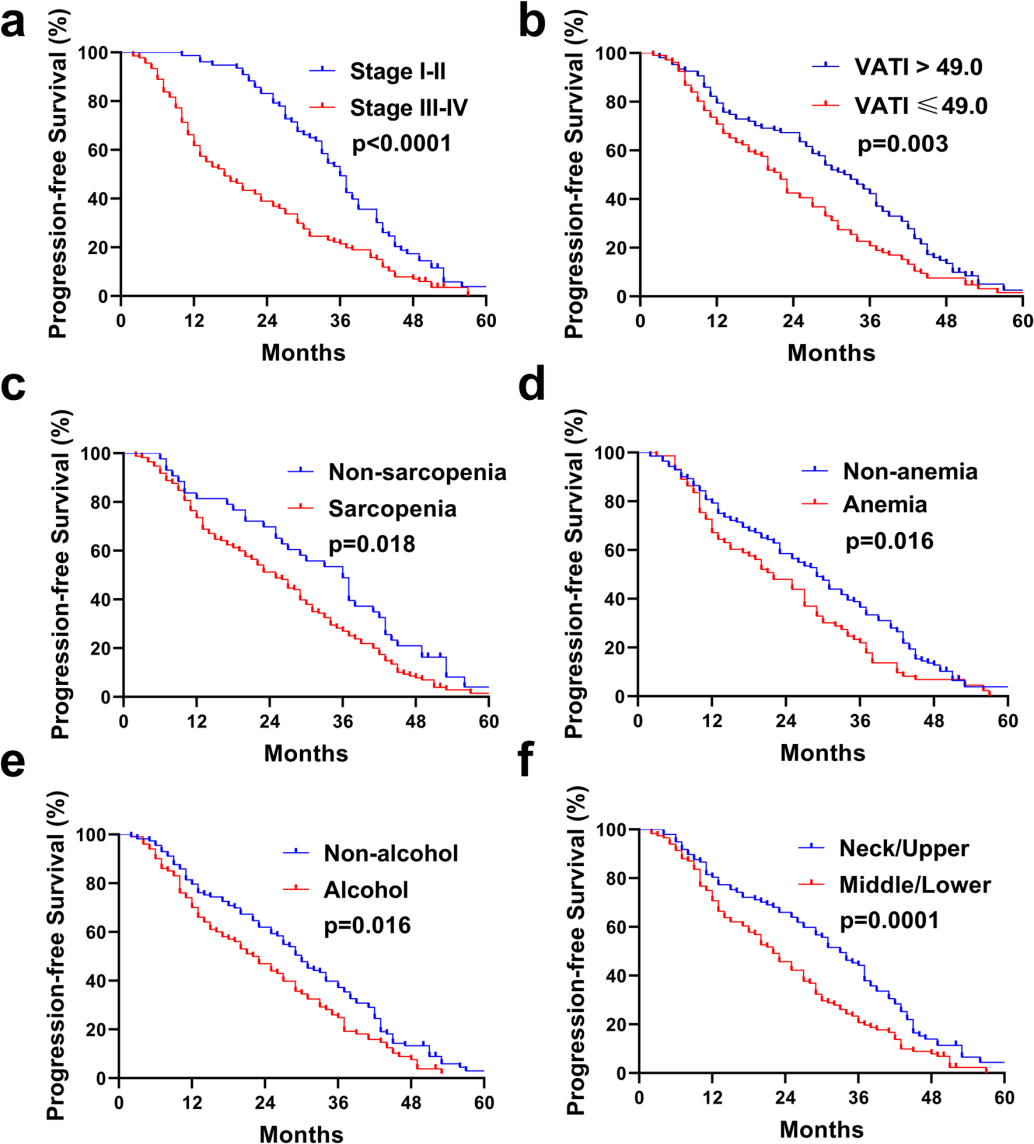
Kaplan–Meier survival curves. (**A**) PFS of patients according to TNM stage. (**B**) PFS of patients with VATI ≤ 49 or > 49. (**C**) PFS of patients according to sarcopeina. (**D**) PFS of patients according to anemia. (**E**) PFS of patients according to alcohol. (**F**) PFS of patients according to tumor location

The median follow-up duration was 35 (range, 4–77) months. The median OS of the 213 patients was 35 months and the 1-, 3-, and 5-year OS rates were 93.9%, 49.8%, and 9.4%, respectively. The 5-year OS in the sarcopenia group was significantly shorter (2.3%) than that in the non-sarcopenia group (7.0%; *p* = 0.016; Fig. 4B). The median PFS of the 213 patients was 26 months and the 1-, 3-, and 5-year PFS rates were 77.9%, 32.4%, and 1.9%, respectively. The 3-year PFS in the sarcopenia group was significantly shorter (27.0%) than that in the non-sarcopenia group (48.8%; *p* = 0.018; Fig. 5C).

### Univariate and multivariate analyses of OS of 213 patients

In the univariate analysis (Table 2), nutritional parameters including serum albumin ≥ 40 g/L, non-sarcopenia, TNM stage I-II and N stage 0–1 were strongly associated with an improved OS. Non-alcohol, NRI ≥ 100, non-anemia, TATI > 83.0, SATI > 27.8, VATI > 49.0, cervical and upper-thoracic locations and T stage 1–2 were the clinicopathological and prognostic factors for longer OS.

**Table 2:** Univariate and multivariate analyses of OS

Characteristic	Univariate			Multivariate		
	HR	95% CI	*p* value	HR	95% CI	*p* value
Age (≥ 65 vs. < 65)	1.104	0.830–1.467	0.497			
Sex (male vs. female)	1.183	0.858–1.632	0.305			
Smoking (yes vs. no)	1.080	0.816–1.428	0.590			
Alcohol (yes vs. no)	1.372	1.036–1.817	**0.027**			
BMI (≥ 24 vs. < 24)	0.750	0.552–1.017	0.064			
NRI (≥ 100 vs. < 100)	0.610	0.426–0.874	**0.007**			
Anemia (yes vs. no)	1.439	1.073–1.928	**0.015**			
Albumin (≥ 40 vs. < 40)	0.582	0.416–0.813	**0.002**	0.658	0.470–0.923	**0.023**
Sarcopenia (yes vs. no)	1.457	1.024–2.074	**0.036**	1.638	1.113–2.410	**0.016**
TATI (> 83.0 vs. ≤ 83.0)	0.689	0.520–0.913	**0.010**			
SATI (> 27.8 vs. ≤ 27.8)	0.707	0.533–0.937	**0.016**			
VATI (> 49.0 vs. ≤ 49.0)	0.659	0.497–0.872	**0.004**			
Location (middle/lower vs. neck/upper)	1.698	1.203–2.125	**0.001**			
Length (≤ 4 vs. > 4)	1.054	0.797–1.394	0.710			
Treatment (CRT vs. RT)	0.275	0.615–1.148	0.841			
RT dose (≤ 60 vs. > 60)	1.136	0.854–1.512	0.382			
TNM stage (III–IV vs. I–II)	1.421	1.226–1.647	**< 0.001**	2.439	1.710–3.228	**< 0.0001**
T stage (T3–4 vs. T1–2)	1.291	1.084–1.539	**0.004**			
N stage (N2–3 vs. N0–1)	1.501	1.120–2.013	**0.007**	2.070	1.124–3.817	**< 0.0001**

*HR* hazard ratio, *CI* confidence interval, *p* values highlighted in bold indicated *p* < 0.05

The median OS (95% CI) in patients with alcohol history was shorter than those without alcohol history [33 versus 43 months, respectively (HR 1.372, 95% CI 1.036–1.817; *p* = 0.027; Table 2)]. Patients with high NRI had better survival rates than those with low NRI [41 versus 32 months, respectively (HR 0.610, 95% CI 0.426–0.874; *p* = 0.007)]. Patients with anemia had a shorter time than those with non-anemia [35 versus 41 months, respectively (HR 1.439, 95% CI 1.073–1.928; *p*** = **0.015)]. Patients with high serum albumin had better survival rates than those with low serum albumin [41 versus 32 months, respectively (HR 0.582, 95% CI 0.416–0.813; *p* = 0.002; Table 2, Fig. 4A)]. The median OS in patients in the non-sarcopenia group was longer than that in the patients in the sarcopenia group [47 versus 35 months, respectively (HR 1.457, 95% CI 1.024–2.074; *p* = 0.036; Fig. 4B)]. Patients with high TATI had better survival rates than those with low TATI [45 versus 32 months, respectively (HR 0.689, 95% CI 0.520–0.913; *p* = 0.01)]. Patients with high SATI had a longer time than those with low SATI [45 versus 33 months, respectively (HR 0.707, 95% CI 0.533–0.937; *p*** = **0.016)]. Patients with high VATI had a longer time than those with low VATI [45 versus 32 months, respectively (HR 0.659, 95% CI 0.497–0.872; *p*** = **0.004)]. Patients with N stage 2–3 ESCC had a shorter time than those with stage 0–1 [29 versus 48 months, respectively (HR 1.501, 95% CI 1.120–2.013; *p*** = **0.007; Fig. 4C)]. Patients with TNM stage III–IV ESCC had a shorter time than those with stage I–II [28 versus 51 months, respectively (HR 1.421, 95% CI 1.226–1.647; *p*** < **0.001; Fig. 4D)]. Patients with T stage 3–4 ESCC had a shorter survival time than those with stage 1–2 [34 versus 51 months, respectively (HR 1.291, 95% CI 1.084–1.539; *p* = 0.004)]. Patients with middle and lower-thoracic ESCC lived for a shorter OS time than those with cervical and upper-thoracic ESCC [33 (29.35–36.65) months versus 47 (41.57–52.43) months, respectively (HR 1.698, 95% CI 1.203–2.125; *p* = 0.001)]. Multivariate analysis (Table 2) revealed that serum albumin [HR 0.658 (0.470–0.923); *p* = 0.023], sarcopenia [HR 1.638 (1.113–2.410); *p* = 0.016], TNM stage [HR 2.439 (1.710–3.228); *p* < 0.0001], and N stage [HR 0.483 (0.262–0.890); *p* < 0.0001] were independent prognostic factors for OS in ESCC patients.

### Univariate and multivariate analyses of PFS of 213 patients

Univariate analysis (Table 3) of predictive factors of PFS showed that non-alcohol history, non-anemia, non-sarcopenia, VATI > 49, cervical and upper-thoracic locations and TNM stage I-II were strongly correlated with a longer PFS. NRI ≥ 100, serum albumin ≥ 40 g/L, TATI > 83, SATI > 27.8, T stage 1–2 and N stage 0–1 were the clinicopathological features that predicted a better PFS.

**Table 3:** Univariate and multivariate analyses of PFS

Characteristic	Univariate			Multivariate		
	HR	95% CI	*p* value	HR	95% CI	*p* value
Age (≥ 65 vs. < 65)	1.126	0.847–1.496	0.414			
Sex (male vs. female)	1.187	0.861–1.639	0.295			
Smoking (yes vs. no)	1.093	0.827–1.446	0.531			
Alcohol (yes vs. no)	1.401	1.056–1.859	**0.019**	1.539	1.144–2.070	**0.016**
BMI (≥ 24 vs. < 24)	0.746	0.550–1.011	0.059			
NRI (≥ 100 vs. < 100)	0.614	0.429–0.879	**0.008**			
Anemia (yes vs. no)	1.416	1.057–1.898	**0.020**	1.428	1.043–1.954	**0.016**
Albumin (≥ 40 vs. < 40)	0.589	0.421–0.822	**0.002**			
Sarcopenia (yes vs. no)	1.546	1.086–2.202	**0.016**	1.509	1.052–2.164	**0.018**
TATI (> 83.0 vs. ≤ 83.0)	0.671	0.506–0.889	**0.005**			
SATI (> 27.8 vs. ≤ 27.8)	0.690	0.521–0.914	**0.010**			
VATI (> 49.0 vs. ≤ 49.0)	0.662	0.500–0.877	**0.004**	0.667	0.497–0.895	**0.003**
Location (middle/lower vs. neck/upper)	1.601	1.205–2.129	**0.001**	1.352	1.008–1.812	**0.001**
Length (≤ 4 vs. > 4)	1.042	0.788–1.378	0.772			
Treatment (CRT vs. RT)	0.838	0.619–1.133	0.251			
RT dose (≤ 60 vs. > 60)	1.158	0.870–1.542	0.314			
TNM stage (III–IV vs. I–II)	1.419	1.225–1.645	**< 0.001**	1.384	1.193–1.605	**< 0.0001**
T stage (T3–4 vs. T1–2)	1.269	1.066–1.510	**0.007**			
N stage (N2–3 vs. N0–1)	1.492	1.113–2.001	**0.008**			

*HR* hazard ratio, *CI* confidence interval, *p* values highlighted in bold indicated *p* < 0.05

In the multivariate analysis (Table 3), Patients with TNM stage III–IV ESCC lived for a shorter PFS time than those with stage I–II [17 versus 36 months, respectively (HR 1.384, 95% CI 1.193–1.605; *p* < 0.0001; Fig. 5A)]. Patients with VATI > 49.0 had better survival rates than those with low VATI ≤ 49.0 [33 versus 22 months, respectively (HR 0.667, 95% CI 0.497–0.895; *p* = 0.003; Fig. 5B)]. The median PFS in patients in the non-sarcopenia group was longer than that in patients in the sarcopenia group [36 (29.58*–*42.43) months versus 25 (21.20–28.80) months, respectively (HR 1.509, 95% CI 1.052–2.164; *p* = 0.018; Fig. 5C)]. Patients of ESCC with anemia had a shorter PFS time than those with non-anemia [22 versus 29 months, respectively (HR 1.428, 95% CI 1.043–1.954; *p* = 0.016; Fig. 5D)]. Patients of ESCC with alcohol history had a shorter PFS duration than those with non-alcohol history [22 versus 30 months, respectively (HR 1.539, 95% CI 1.144–2.070; *p* = 0.016; Fig. 5E)]. Patients with middle and lower-thoracic ESCC had a shorter PFS duration than those with cervical and upper-thoracic ESCC [22 versus 33 months, respectively (HR 1.352, 95% CI 1.008–1.812; *p* = 0.0001; Fig. 5F)].

## Discussion

Our study demonstrated that sarcopenia, serum albumin < 40 g/L, N stage 2–3 and TNM stage III–IV are independently associated with poor OS in 213 ESCC patients who received RT or CRT in this analysis. The predictors of sarcopenia can be identified easily on any preoperative computer tomography images and might be helpful in the difficult triage before those treatments.

Cancer patients are likely to have severe nutrition and metabolism problems prior to any treatment [26]. Malnutrition has been suggested to be associated with possible mechanisms including abnormal protein and energy metabolism of tumor cells and aberrant inflammation and immunity, as well as cancer-related symptoms, such as fatigue, pain, coughing and loss of appetite. Malnutrition has been more prevalent among ESCC patients, of whom approximately 60% experienced baseline nutrition problems [22, 23, 25]. Our results agreed well with those previous studies. Sarcopenia is a common index reflecting systemic nutritional status of patients due to cancer progression [7, 27]. A similar study conducted by Mallet et al. [2] found that sarcopenia was an independent predictor of shorter OS in patients with locally advanced esophageal cancer. The research demonstrated that, besides sarcopenia, other factors including BMI, and NRI were significant but not independent predictors of OS in patients with locally advanced esophageal cancer. The difference between our and Mallet’s study is that pathology type of tumor in patients we selected was ESCC considering the heterogeneity of subtypes of esophageal cancer. Furthermore, other nutritional factors including hemoglobin, SATI, VATI, and TATI were calculated and compared with sarcopenia, which was not performed in previous study mentioned above. The results of our study showed that sarcopenia, together with albumin, TNM stage and N stage, were independent predictors for OS in ESCC patients who received RT or CRT. In patients stratified by the sarcopenia index, variables including age, gender, smoking, alcohol, BMI, NRI, anemia, albumin, VATI, TATI, primary tumor locations, tumor length, treatment strategies, TNM stage, T stage, and N stage were similar between the two subgroups. Sarcopenia was significantly associated with lower SATI.

Considering the fact that the etiology of sarcopenia is currently known to be multifactorial, with contributions from systemic inflammation, reduced nutrient intake and activity levels, and increased metabolic rate [21, 24, 28], this would serve as an explanation why sarcopenia correlate with lower SATI, which usually results from the similar malnutritional factors. In this study, our findings verified the prognostic value of sarcopenia as an independent predictor of both OS and PFS in ESCC patients who received RT or CRT. Baseline nutritional status was usually evaluated with systemic inflammatory reaction biomarkers and relative parameters [2]. Studies have shown that serum albumin is related to the prognosis of cancer patients [29, 30]. Our results suggested that albumin was a predictor of OS in the univariate and multivariate analysis that was performed in this study. It could be inferred that albumin may be useful in reflecting patients’ physical performance, which was possibly a crucial premise of optimal treatment and better clinical outcomes. However, some studies also suggested hypoalbuminemia might be more related to hydration or inflammation status than to malnutrition [26, 31]. In addition, alcohol, NRI < 100, anemia, VATI ≤ 49, TATI ≤ 83 and T stage 3–4 were significant predictor of poorer OS and PFS, respectively. Interestingly, no significant correlation between alcohol and sarcopenia was observed in this study despite of the high significance of sarcopenia in ethanol-associated cirrhosis since alcohol could mediate dysregulation of protein homeostasis and then leaded to sarcopenia [32]. The reason might be due to confounding effects of cancer itself and treatment. Primary tumor location was stratified into two groups in our study: (1) cervical and upper-thoracic ESCC; and (2) middle-thoracic and lower-thoracic ESCC. The primary tumor location is also important factor affecting survival outcomes. Our results suggested that primary tumor location was significant predictor of OS in the univariate analysis, and independent predictor of PFS. Patients with middle and lower-thoracic ESCC had impaired PFS and OS, which was similar with another study [33]. It could be inferred that patients with middle-thoracic and lower-thoracic ESCC may be more prone to malnutritional status due to reductions in nutrient intake resulted from esophageal obstruction. Our results also suggested that TNM stage was an independent predictor of both OS and PFS in this study. As expected, ESCC patients with TNM stage III-IV had shorter OS and PFS, which was comparable to those of previous studies on patients with ESCC who were treated with definitive radiotherapy [34]. N stage was also an independent prognostic factor of OS in this study, which agreed well with previous studies on patients with ESCC treated with definitive CRT [35].

There are limitations of present study related to somewhat smaller population in a single center, who received radiotherapy combined with chemotherapy. However, the results of this study still presented the potential of sarcopenia as a tool for predicting survival of patient independent of other factors, including albumin, N stage and TNM stage. Nevertheless, multi-center larger investigations and prospective clinical trials are needed to establish the role of the sarcopenia nutritional index for predicting clinical outcomes.

In conclusion, sarcopenia and other indexes of body composition such as lower serum albumin may be valuable to identify potential nutritional problems and poorer survival in our study. They are easily conducted on any preoperative computer tomography scan or blood examination and might be useful in patient risk stratification and help oncologists provide optimal intervention strategy before treatment initiation.

## Data Availability

The data supporting the findings of this study are available from the corresponding author upon reasonable request.

## Abbreviations

PFS: Progression free survival
OS: Overall survival
ESCC: Esophageal squamous cell cancer
CRT: Chemoradiotherapy
BMI: Body mass index
SATI: Subcutaneous adipose tissue index
VATI: Visceral adipose tissue index
TATI: Total adipose tissue index
SMI: Skeletal muscle index

## References

[1] Tamandl D, Paireder M, Asari R (2016). Markers of sarcopenia quantified by computed tomography predict adverse long-term outcome in patients with resected oesophageal or gastro-oesophageal junction cancer. Eur Radiol.

[2] Mallet R, Modzelewski R, Lequesne J (2020). Prognostic value of sarcopenia in patients treated by radiochemotherapy for locally advanced oesophageal cancer. Radiat Oncol.

[3] Lordick F, Mariette C, Haustermans K (2016). Oesophageal cancer: ESMO Clinical Practice Guidelines for diagnosis, treatment and follow-up. Ann Oncol.

[4] Zhang AD, Su XH, Shi GF (2020). Survival comparision of three-dimensional radiotherapy alone vs. chemoradiotherapy for esophageal squamous cell carcinoma. Arch Med Res.

[5] Fearon K, Strasser F, Anker SD (2011). Definition and classification of cancer cachexia: an international consensus. Lancet Oncol.

[6] Anandavadivelan P, Lagergren P (2016). Cachexia in patients with oesophageal cancer. Nat Rev Clin Oncol.

[7] Hagens ERC, Feenstra ML, van Egmond MA (2020). Influence of body composition and muscle strength on outcomes after multimodal oesophageal cancer treatment. J Cachexia Sarcopenia Muscle.

[8] Cox S, Powell C, Carter B (2016). Role of nutritional status and intervention in oesophageal cancer treated with definitive chemoradiotherapy: outcomes from SCOPE1. Br J Cancer.

[9] Lee JLC, Leong LP, Lim SL (2016). Nutrition intervention approaches to reduce malnutrition in oncology patients: a systematic review. Support Care Cancer.

[10] Chen LK, Woo J, Assantachai P (2020). Asian Working Group for Sarcopenia: 2019 consensus update on sarcopenia diagnosis and treatment. J Am Med Dir Assoc.

[11] Wong A, Zhu D, Kraus D (2021). Radiologically defined sarcopenia affects survival in head and neck cancer: a meta-analysis. Laryngoscope.

[12] Yang M, Shen Y, Tan L (2019). Prognostic value of sarcopenia in lung cancer: a systematic review and meta-analysis. Chest.

[13] Buentzel J, Heinz J, Bleckmann A (2019). Sarcopenia as prognostic factor in lung cancer patients: a systematic review and meta-analysis. Anticancer Res.

[14] Choi MH, Oh SN, Lee IK (2018). Sarcopenia is negatively associated with long-term outcomes in locally advanced rectal cancer. J Cachexia Sarcopenia Muscle.

[15] Olmez T, Ofluoglu CB, Sert OZ (2020). The impact of sarcopenia on pathologic complete response following neoadjuvant chemoradiation in rectal cancer. Langenbecks Arch Surg.

[16] Kuwada K, Kuroda S, Kikuchi S (2019). Clinical impact of sarcopenia on gastric cancer. Anticancer Res.

[17] Hu X, Liao DW, Yang ZQ (2020). Sarcopenia predicts prognosis of patients with renal cell carcinoma: a systematic review and meta-analysis. Int Braz J Urol.

[18] Watanabe A, Oshikiri T, Sawada R, et al., Actual sarcopenia reflects poor prognosis in patients with esophageal cancer*.* Ann Surg Oncol. 2022. 10.1245/s10434-022-11337-2 35169977

[19] Mourtzakis M, Prado CM, Lieffers JR (2008). A practical and precise approach to quantification of body composition in cancer patients using computed tomography images acquired during routine care. Appl Physiol Nutr Metab.

[20] Sato S, Demura S, Nakai M (2015). Storage capacity of subcutaneous fat in Japanese adults. Eur J Clin Nutr.

[21] Huang CH, Lue KH, Hsieh TC, Liu SH, Wang TF, Peng TC (2020). Association between sarcopenia and clinical outcomes in patients with esophageal cancer under neoadjuvant therapy. Anticancer Res.

[22] Chen S, Cao R, Liu C (2020). Investigation of IL-4, IL-10, and HVEM polymorphisms with esophageal squamous cell carcinoma: a case-control study involving 1929 participants. Biosci Rep.

[23] Guo J, Zheng C, Xiao Q (2015). Impact of anaemia on lung function and exercise capacity in patients with stable severe chronic obstructive pulmonary disease. BMJ Open.

[24] Zhao J, Lei T, Zhang T (2020). The efficacy and safety of simultaneous integrated dose reduction in clinical target volume with intensity-modulated radiotherapy for patients with locally advanced esophageal squamous cell carcinoma. Ann Transl Med.

[25] Fujiya K, Kawamura T, Omae K (2018). Impact of malnutrition after gastrectomy for gastric cancer on long-term survival. Ann Surg Oncol.

[26] Bouillanne O, Morineau G, Dupont C (2005). Geriatric Nutritional Risk Index: a new index for evaluating at-risk elderly medical patients. Am J Clin Nutr.

[27] Shichinohe T, Uemura S, Hirano S (2019). Impact of preoperative skeletal muscle mass and nutritional status on short-and long-term outcomes after esophagectomy for esophageal cancer: a retrospective observational study: impact of psoas muscle mass and body mass on esophagectomy. Ann Surg Oncol.

[28] Tan BH, Brammer K, Randhawa N, Welch NT, Parsons SL, James EJ, Catton JA (2015). Sarcopenia is associated with toxicity in patients undergoing neo-adjuvant chemotherapy for oesophago-gastric cancer. Eur J Surg Oncol.

[29] Digant Gupta CGL (2010). Pretreatment serum albumin as a predictor of cancer survival: a systematic review of the epidemiological literature. Nutr J.

[30] Shen Y, Li H, Yuan ZQ (2020). Low pretreatment PNI correlates with worse survival in patients with stage III/IV NSCLC who received chemotherapy. Neoplasma.

[31] Ballmer PE (2001). Causes and mechanisms of hypoalbuminaemia. Clin Nutr.

[32] Davuluri G, Welch N, Sekar J (2021). Activated protein phosphatase 2A disrupts nutrient sensing balance between mechanistic target of rapamycin complex 1 and adenosine monophosphate-activated protein kinase, causing sarcopenia in alcohol-associated liver disease. Hepatology.

[33] Hironaka S, Komori A, Machida R (2020). The association of primary tumor site with acute adverse event and efficacy of definitive chemoradiotherapy for cStage II/III esophageal cancer: an exploratory analysis of JCOG0909. Esophagus.

[34] Inada M, Nishimura Y, Ishikawa K (2019). Comparing the 7th and 8th editions of the American Joint Committee on Cancer/Union for International Cancer Control TNM staging system for esophageal squamous cell carcinoma treated by definitive radiotherapy. Esophagus.

[35] Zhao Z, Zhang Y, Wang P (2019). The impact of the nodal status on the overall survival of non-surgical patients with esophageal squamous cell carcinoma. Radiat Oncol.

